# Short Report of Longitudinal CGRP-Measurements in Migraineurs During a Hypoxic Challenge

**DOI:** 10.3389/fneur.2022.925748

**Published:** 2022-07-28

**Authors:** Florian Frank, Katharina Kaltseis, Karl Messlinger, Gregor Broessner

**Affiliations:** ^1^Department of Neurology, Headache Outpatient Clinic, Medical University of Innsbruck, Innsbruck, Austria; ^2^Institute of Physiology and Pathophysiology, Friedrich-Alexander-University Erlangen-Nürnberg, Erlangen, Germany

**Keywords:** CGRP, longitudinal measurement, plasma levels, migraine, headache, hypoxia

## Abstract

**Background:**

Calcitonin gene related peptide (CGRP) plays a key role in the pathophysiology of migraine and is therefore considered a potential biomarker for primary headache disorders. The challenge remaining is establishing standardized protocols for its assessment in various extracellular compartments and identifying pathological situations associated with an increase in CGRP.

**Methods:**

We performed longitudinal measurements of CGRP plasma levels in 30 volunteers with the diagnosis of episodic migraine with and without aura under controlled circumstances during an induced migraine attack under a hypoxic challenge. Blood samples were collected from a cubital vein and CGRP plasma levels measured using ELISA.

**Results:**

CGRP levels varied significantly between the subjects at baseline (15.48–1,889.31 pg/ml) but were neither associated with socio-demographic data nor with headache/migraine frequency or intensity collected before hypoxic exposure. CGRP levels during hypoxia fluctuated around baseline and increased with prolonged hypoxia but did not differ significantly in subjects with migraine or headache compared to those without. However, subjects experiencing migraine without aura showed significantly higher levels than those with aura. Ictal CGRP levels were increased in females, in subjects with a negative family history regarding headaches, in those older than 30 years of age or with a recent headache attack before the experiment (*p* < 0.05).

**Conclusion:**

CGRP plasma levels seem to be highly variable even at baseline in migraine patients and increased during hypoxic challenge and migraine attacks. This is the first in human longitudinal measurement of peripheral CGRP levels during induced migraine attacks using a highly standardized protocol.

## Introduction

The debilitating nature and socio-economic consequences of migraine have increased the interest in developing therapies directly targeting the pathophysiology of migraine generation. Hence, patients now have access to drugs interfering with the calcitonin gene-related peptide (CGRP) pathway. This 37 amino-acid neuropeptide was shown to play a major role in the pathogenesis of migraine (1). Besides its potent vasodilatory effect, CGRP contributes to neurogenic inflammatory responses and possibly to sensitization of trigeminal nociceptors when it is released in cranial tissues. Together with glutamate, CGRP is also released, in parallel with substance P, in the spinal trigeminal nucleus, where it contributes to central sensitization and enhancement of nociceptive transmission (2). After its release, CGRP is degraded within minutes by peptidases resulting in significantly reduced concentrations in the peripheral circulatory system.

Since CGRP has been attributed a pivotal function in the trigeminovascular system, research has focused on its pathophysiologic significance in pain disorders such as migraine and other primary and secondary headaches. Migraine and many of its mimics are diagnosed solely through patient reported medical history utilizing the diagnostic criteria of the International Classification of Headache Disorders, version 3 (ICHD-III) (3).

Whereas other disease entities can be confirmed through standardized laboratory tests, a well-established surrogate marker for primary headache disorders has yet to be developed. In line with several previous approaches, it appears consequent to assess easy-to-measure peripheral CGRP levels as biomarker in headache disorders (4, 5). In addition, from a clinical point of view, it would be extremely desirable to have an easily accessible parameter that objectifies or even predicts the response to costly therapies such as monoclonal antibodies targeting CGRP or its receptor.

There are some inherent caveats in the development of CGRP as a biomarker. First and foremost, the short half-life of about 10 mins as well as the still not entirely understood abundance of possible sites of production and elimination as well as cross-interaction between CGRP subtypes and CGRP-receptor subtypes and associated receptors add to the complexity of establishing such a marker (6, 7).

Another limitation is the heterogenous approach to CGRP extraction and measurement across different research groups. This has recently been addressed by a profound methodological study on CGRP measurement in peripheral human blood (8). Based on this study, we analysed ictal CGRP concentrations of migraineurs that were exposed to a hypoxic challenge that triggers migraine headaches.

The rationale to use hypoxia as a trigger for migraine headaches derives from multiple observations. These include higher prevalence of migraines in elevated regions (9, 10) oxidative stress as mechanism for migraine triggers (11), detection of tissue hypoxia during cortical spreading depression (12) and, foremost, safe and successful induction of migraine headache in experimental settings utilizing hypoxia (13). The hypoxic challenge performed in this study has been described in an earlier publication of our group (14).

## Methods

### Sample Characteristics

Thirty volunteers were recruited from our tertiary headache outpatient clinic and *via* advertisement at the Medical University of Innsbruck. The study was approved by the local ethics community (AN2016-0126 363/4.14).

Participants met the following inclusion criteria: diagnosis of migraine with or without aura according to the ICHD-3 diagnostic criteria, history of migraine for over 12 months and migraine frequency of at least 1 day per month over the last 3 months prior to screening. Participants with chronic migraine and/or medication overuse were excluded, as well as patients who received preventative migraine treatment (e.g., betablockers, antiepileptics, tricyclic antidepressants etc.) during 12 months prior to the screening. If a participant reported headaches or used acute medication within 24 h before the experiment, it was postponed. Eligible patients had to complete a headache diary 10 days prior to and 10 days after the experiment. The trial was conducted in a normobaric hypoxic chamber (NHC) located on the campus of the University of Innsbruck's Department of Sport Science (590 m). The inspiratory fraction of oxygen (FiO_2_) in the NHC was lowered to 12.6% to simulate a stay at 4,500 m above sea level. A detailed description of the methods and the experimental design have been published elsewhere (14). All volunteers entered the NHC at ~9.00 am to avoid possible circadian fluctuations of CGRP. The use of acute medication to alleviate the headaches was not permitted at any time during the experiment.

### Sample Collection

First blood samples were drawn at the beginning of the experiment prior to the hypoxic challenge (T0). Consecutive blood samples were taken under hypoxia at hourly intervals after entering the NHC (T1-Toff). After 6 h, the experiment in the NHC was terminated and 2 follow-up blood samples after 1 and 2 h post hypoxic exposure were taken. Blood was drawn from a cubital vein using EDTA-K tubes (S-Monovetten, Sarstedt, Nümbrecht, Germany) and centrifuged at 4°C for ~4 min with 4,000 rpm. The supernatant plasma was taken off with an Eppendorff pipette, transferred to cryovials (Nunc CryoTubes, Merck, Darmstadt, Germany), and frozen at −80°C within 10–12 min.

### Sample Processing

For detailed information on the processing and analysis of the samples, please refer to our previous article (8). In short, samples were processed with a double-antibody sandwich enzyme-linked immunosorbent assay (ELISA; CGRP Enzyme Immunoassay #A05481, shortly named CGRP EIA, Bertin Bioreagent, Montigny-le-Bretonneux, France) for α- and β-CGRP, with a cross-reactivity with amylin, calcitonin and substance *P* of <0.01%. For this purpose, a synthetic interstitial solution was prepared, and protease inhibitors were added to create a standard buffer, which was fitted with human CGRP and diluted to create serial dilutions of CGRP. Furthermore, human blood plasma was used as an alternative to the standard buffer. With these preparations a reference curve was fitted to later determine the individual CGRP concentrations of each sample.

### Data Analysis

The Shapiro-Wilk-Test was used to test for normality. To reduce skewness, we applied the log-transformation χ (15). We performed a repeated measures ANOVA to determine the changes of the CGRP levels over the time points. The Greenhouse–Geisser adjustment was used to correct for violations of sphericity. To test for possible vulnerability, the Pearson's correlation coefficient was used to associate CGRP levels with periinterventional headache/migraine days as well as hours since the last headache/migraine attack before the experiment. A *p* value of 0.05 was considered significant. Values are given as mean ± standard error of the mean (SEM) or as median with confidence intervals (box plots). Missing data on follow-up observation was addressed by using the last observation carried forward (LOCF) principle. The analyses were performed with SPSS version 26.0 (IBM Corporation, Armonk, NY, US).

## Results

### Sample Characteristics

We assessed plasma samples of a total of 30 patients (22 female, 8 male) with episodic migraine according to ICHD-3 diagnostic criteria, of which 16 patients (11 women) had migraine with aura and 14 patients (11 women) had migraine without aura. The examinations were carried out for 26 patients per protocol, enduring 6 h under hypoxia. Three participants left the NHC prematurely, due to severe migraine headache, and one examination was discontinued for safety reasons due to a pronounced decrease in systolic blood pressure in one asymptomatic patient. All participants were followed up 24 h after leaving the NHC. Mean age was 27.56 years (SD ± 7.54), 24 (80%) were younger than 30 years. Mean body mass index was 21.74 kg/m^2^ (SD ± 2.63). Mean monthly migraine attack frequency, as reported by the patients, was 3.25 attacks (SD ± 3.05). Mean monthly intake of abortive migraine medication was 3.39 days (SD ± 5.88). Twenty patients (66.7%) had a positive family history for migraine (Table 1).

**Table 1:** Baseline characteristics of participants allocated to headache-type group under hypoxia.

**Characteristic**	**Headache**			**Migraine**			**Aura**			**Total**
	**Headache *n* =24 (80.0%)**	**No headache *n* = 6 (20.0%)**	***p*-value**	**Migraine *n* = 19 (63.3%)**	**No migraine n=11 (36.6%)**	***p*-value**	**Aura *n* = 5 (16.6%)**	**No aura *n* = 25 (83.3%)**	***p*-value**	***n* = 30**
Female, *n* (%)	19 (63.3%)	3 (10.0%)	0.175	14 (46.6%)	8 (26.6%)	0.637	3 (10.0%)	19 (63.3%)	0.405	22 (73.3%)
Male, *n* (%)	5 (16.6%)	3 (10.0%)		5 (16.6%)	3 (10.0%)		2 (6.6%)	6 (20.0%)		8 (26.6%)
Age, years ± SD	26.5 ± 6.8	31.8 ± 3.8	0.125	27.1 ± 7.5	28.3 ± 7.9	0.684	32.2 ± 9.6	26.6 ± 6.9	0.135	27.6 ± 7.5
BMI, kg/m^2^ ± SD	21.3 ± 2.4	23.4 ± 2.4	0.077	21.2 ± 2.5	22.7 ± 2.7	0.140	22.4 ± 2.7	21.6 ± 2.6	0.015	21.7 ± 2.6
Monthly migraine attack frequency, days ± SD	3.6 ± 3.2	1.7 ± 1.7	0.171	4.1 ± 3.5	1.8 ± 1.4	0.053	6.4 ± 5.3	2.6 ± 2.0	0.580	3.2 ± 3.0
Monthly intake of acute medication, days ± SD	3.6 ± 6.4	2.3 ± 1.9	0.680	3.8 ± 6.9	2.3 ± 1.4	0.565	9.5 ± 13.7	2.2 ± 1.7	0.363	3.4 ± 5.9
Prior use of migraine prophylaxis, *n* (%)	7 (100%)	0 (0%)	NA	7 (100%)	0 (0%)	NA	2 (28.6%)	5 (71.4%)	NA	7 (23.3%)
Family history of migraine, *n* (%)	17 (85.0%)	3 (15.0%)	0.372	14 (70.0%)	6 (30.0%)	0.425	4 (20.0%)	16 (80.0%)	0.640	20 (66.7%)

*Table modified from “Migraine and aura triggered by normobaric hypoxia” by Frank et al. (14)*.

*Two-sided Fisher's exact-test, with Yate's correction when appropriate, was carried out to compare continuous means between groups. Categorial data was analysed for correlation using Chi^2^-test. Level of statistical significance was defined as α = 5% (p-value = 0.05)*.

*NA, not applicable*.

### Headache and Migraine

A total of 24 patients (80.0%) reported headaches during the experiment. Nineteen patients (63.3%) developed migraine headache accompanied by autonomic features such as nausea, photophobia and phonophobia, and five (16.7%) developed migraine aura. Incidence of total headache and migraine was increasing throughout the experiment and peaked at Toff, which entailed volunteers completing 6 h of exposition to hypoxia as well as those terminating prematurely. The mean onset of headache was between T4/T5 and that of migraine at T5 during the experiment.

### CGRP

#### CGRP at Baseline

**Figure 1:**
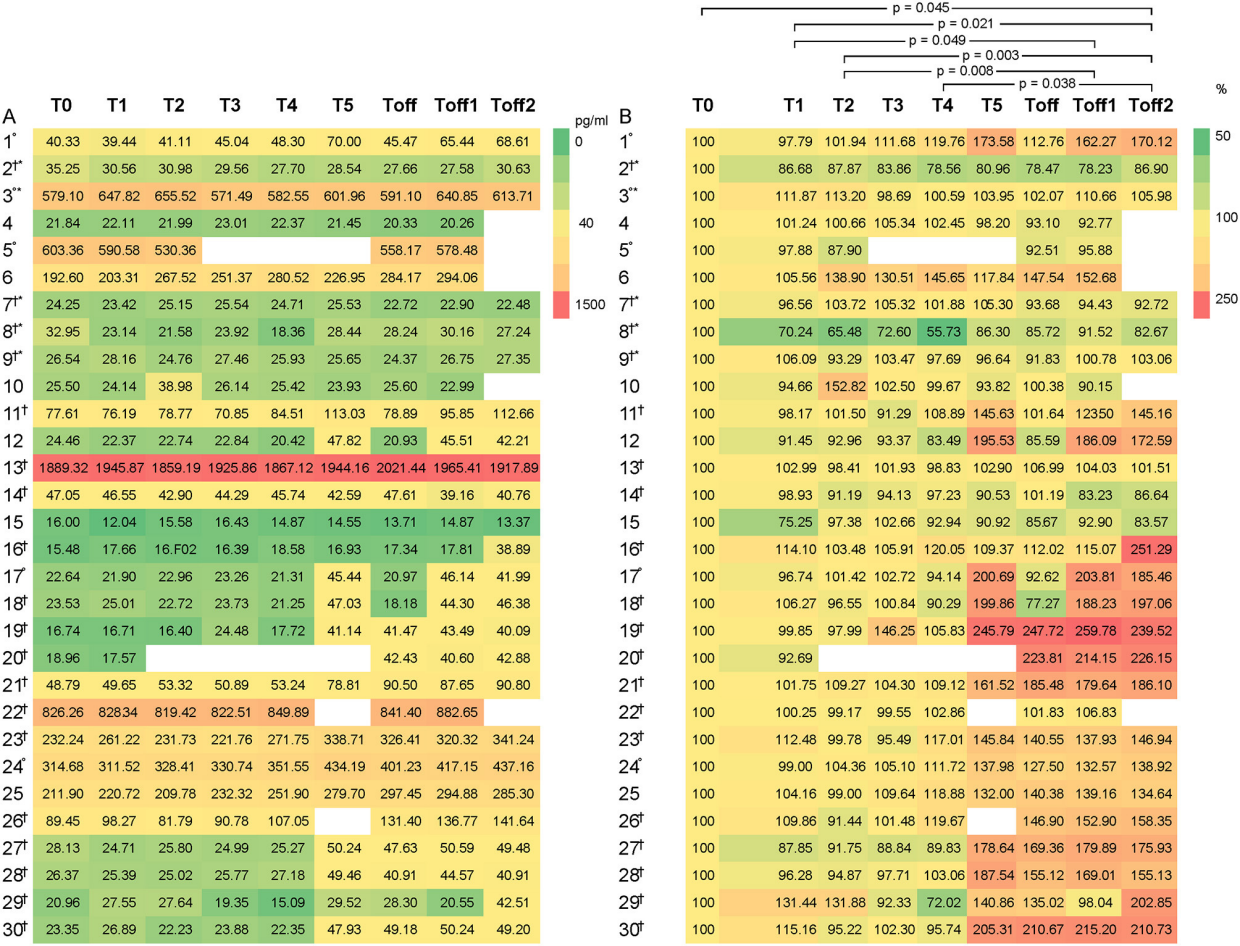
Heat maps to illustrate the distribution of CGRP levels, given as pg/ml, among the participants (1–30). CGRP levels are displayed as colors ranging from green to red as shown in the key. The X-axis shows the different times points of the experiment with T0 as baseline, T1 as first blood sample 1 h after entering the HAC and consecutive hourly blood samples. Toff represents the first blood sample immediately after leaving the HAC. On the left side **(A)**, the absolute values of the CGRP-levels are indicated and on the right side **(B)** the percentage change compared to baseline. Subject experiencing °headache; ^†^migraine; ^*^aura during the experiment. Participant 3 experienced headache that did not fulfill the ICHD criteria for migraine headache due to a lack of nausea or photophobia and phonophobia but had aura symptoms.

CGRP levels differed significantly between the subjects, ranging from 15.48 to 1,889.31 pg/ml at baseline (Figure 1). High CGRP plasma levels at baseline were not associated with age, monthly migraine or headache days, attack frequency, attack duration, attack intensity, sex, family history of migraine, the use of abortive medication, years lived with migraine or headache, BMI, level of physical activity or any other of the collected data. There were two outliers regarding baseline and consecutive CGRP levels, subject 13 (female, 42 years, mean CGRP 1,926 pg/ml) and subject 22 (female, 22 years, mean CGRP 838.64 pg/ml). However, no differences in categorial or metric variables were found for these two participants.

#### CGRP During Hypoxic Challenge

The mean absolute CGRP concentration over all time points showed no significant variation regarding absolute values and standard deviation between the subjects (Table 2).

**Table 2:** Overview of mean CGRP concentration throughout the experiment.

**Time**	**Mean (CGRP) pg/ml**	**SD**
T0	185.19	380.01
T1	190.29	391.33
T2	192.43	390.31
T3	179.81	390.22
T4	183.67	383.77
T5	179.75	388.92
Toff	206.84	400.88
Toff1	212.93	395.67
Toff2	184.21	390.78

*Throughout all individual time points the mean CGRP concentration and standard deviation (SD) did not vary significantly (p > 0.05). The repeated measures ANOVA was corrected for sphericity using the Greenhouse-Geisser correction*.

In Figure 1B, the relative change of CGRP plasma levels in percent at the different sample time points is compared to the baseline values. An apparent difference was found from baseline compared to T5 onwards. We used the Friedman's test and corrected for multiple testing to analyse each individual CGRP concentration for any given time point. We found a significant difference, with an increase of CGRP levels in line with prolonged hypoxia, between T2–Toff1 (*p* = 0.008), T2–Toff2 (*p* = 0.003), T1–Toff1 (*p* = 0.049), T1–Toff2 (*p* = 0.021), T4–Toff2 (*p* = 0.038) and T0–Toff2 (*p* = 0.045), respectively.

**Figure 2:**
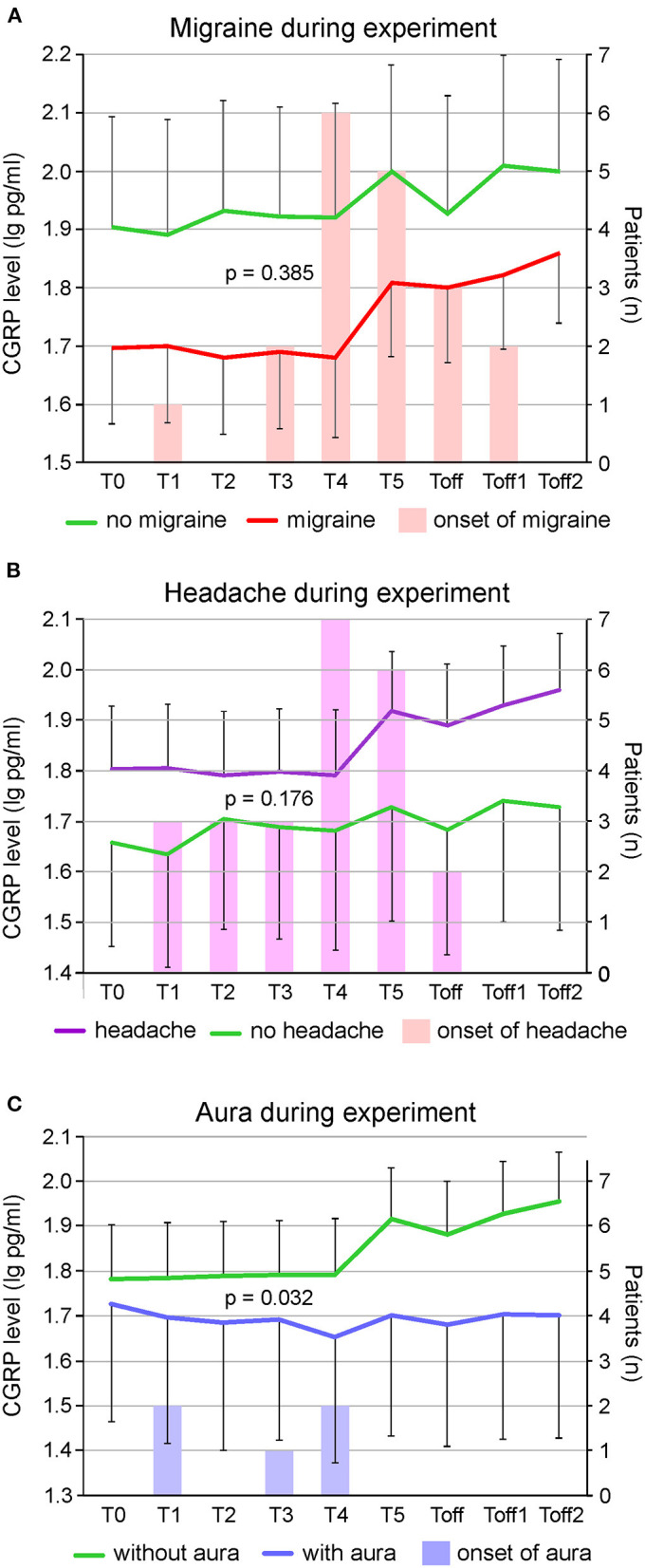
Changes in logarithmized CGRP levels (pg/ml) over the time course of the experiment in participants with migraine (*n* = 24) vs. no migraine (*n* = 6) **(A)**, in subjects with (including migraine, *n* = 19) vs. without headache (*n* = 11) **(B)** and subjects experiencing aura symptoms (*n* = 5) vs. no aura symptoms (*n* = 25) **(C)**. The right Y-axis depicts the number of participants with the onset of migraine **(A)**, headache **(B)** and aura symptoms **(C)** at the different timepoints during the experiment. The lower and upper limits of standard error of the mean are given. There was a significant difference in plasma CGRP levels between subjects reporting aura or not during the experiment (*p* = 0.027) but there were no significant differences in participants with and without migraine (*p* = 0.385) or headache (*p* = 0.176). Mean onset of migraine peaked at T5, of headache between T4 and T5 and for aura at T3.

**Figure 3:**
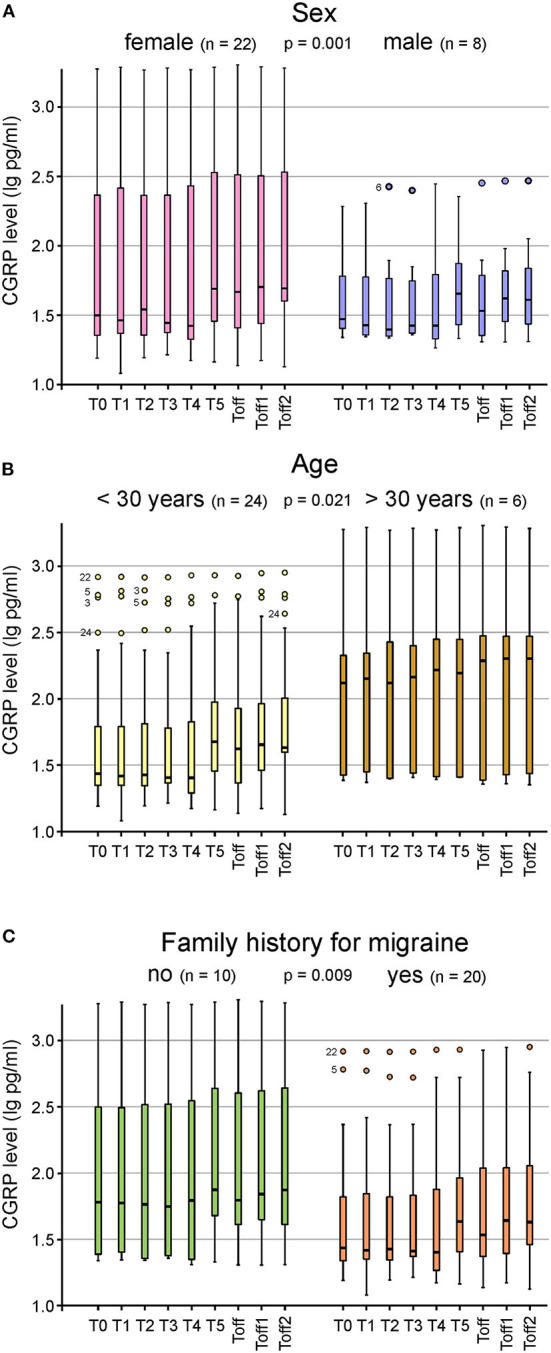
Box plots for the CGRP plasma levels at the different timepoints shown for sex **(A)**, age **(B)** and family history for migraine **(C)**. Significantly higher CGRP levels were found in female subjects (*n* = 22; *p* = 0.001) **(A)**, in participants older than 30 years (*n* = 24; *p* = 0.021) **(B)** and in those with a negative family history (*n* = 10; *p* = 0.009) **(C)**. Mild outliers are marked with a circle (O), the number next to it represents the corresponding patient.

CGRP levels of ictal migraine and headache patients compared to participants with no migraine or headache during the experiment are illustrated in Figures 2A,B. A repeated measures ANOVA with Greenhouse-Geisser correction determined that mean CGRP levels did not show a statistically significant difference between participants experiencing migraine [*F*_(2.73; 76.53)_ = 1.02; *p* = 0.385] or headache [*F*_(2.81; 78.74)_ = 1.70; *p* = 0.176] and subjects with absence of headache or migraine. However, we found a significant difference in CGRP levels between subjects with and without aura during the experiment [*F*_(2.99; 83.69)_ = 3.08; *p* = 0.032] (Figure 2C). Patients experiencing migraine attacks without aura in the course of the hypoxic challenge had significantly higher CGRP concentrations compared to participants with aura symptoms. Higher CGRP levels during the experiment were significantly associated with female sex (*p* = 0.001; Figure 3A), with age (>30 years or <30 years; *p* = 0.021; Figure 3B) and a negative family history of migraine (*p* = 0.009; Figure 3C). No other parameters showed a significant correlation with CGRP concentration (monthly migraine or headache days, headache or migraine intensity, years lived with headache or migraine, BMI, O_2_ saturation, blood pressure, pulse rate).

Using the headache diaries, the time between the last migraine or headache attack and the hypoxic challenge was assessed. Mean temporal lag between the last headache or migraine attack was 96 hours (range 24–240 h) and 132 h (range 24–240 h), respectively. A bivariate Pearson correlation showed a medium correlation between higher CGRP and a shorter lag from the last headache attack (*r* = −0.41; *p* < 0.05), but none for the last migraine attack (*r* = −0.16; *p* = 0.963) or the number of peri-interventional headache days (*r* = 0.282; *p* = 0.139).

## Discussion

Since the 1980s, attempts have been made to quantify CGRP concentration in plasma, saliva, cerebrospinal fluid, or tear fluid (4, 5, 16–20). Due to its short half-life of ~10 mins, lack of standardized procedures for measuring and analysing CGRP as well as the variable sample materials, the study results gathered so far are conflicting. Thus, even 40 years after the discovery of this neuropeptide, still no reference values of ictal and interictal CGRP concentration are determined.

Herein, we present a longitudinal study of a total of 30 participants with episodic migraine with or without aura who were observed under controlled and highly standardized conditions in a normobaric hypoxic chamber. Besides migraine, the volunteers did not suffer from any neurological, psychiatric, cardiovascular, or respiratory disorder. The participants did not overuse acute medication and were not taking any preventative medication for their migraine. The hourly blood samples were taken during a hypoxic challenge. 24 (80%) of the subjects developed a headache, migraine was triggered in 19 (63.3%) and 5 (16.7%) developed a migraine aura. Several studies have identified hypoxia as potent trigger of migraine attacks (14, 21) and therefore hypoxia was used to induce migraine in this study.

Main findings of the study are (1) absolute plasma CGRP concentration differs significantly at baseline without a verifiable explanation. (2) CGRP levels increased significantly in line with hypoxic challenge. (3) A negative family history for migraine, age >30 and female sex were associated with higher CGRP levels during the experiment. (4) Ictal CGRP plasma levels were significantly higher in subjects experiencing migraine without aura. (5) Higher CGRP levels were temporally associated with a recent headache attack.

At baseline CGRP plasma concentration ranged from 15.48 to 1,889.31 pg/ml. We found no explanation for this variation in the variables collected, as there were no significant differences regarding demographic or headache specific features in our subjects. The CGRP concentration remained robust during the experiment intra-individually, indicating stable and standardized testing conditions for each sample, as has been demonstrated before by our group. Therefore, it may be speculated, that CGRP is produced, released and/or degraded at different rates individually.

Hypocapnic hypoxia leads to vasodilatation—a possible involvement of CGRP in the pathophysiology of vasoactive adaptation could be suspected. However, a study did not find altered CGRP levels during hypoxia (22).

In recent years, literature has emerged that offers contradictory results about CGRP measurements in plasma interictally as well as during an attack. Cernuda-Morollón et al. (5) found significantly increased CGRP levels interictally in women with chronic migraine compared to healthy controls or women with a diagnosis of episodic migraine or cluster headache. Fekrazad et al. (23) corroborated these results with their study and consequently proposed the use of CGRP as a biomarker in chronic migraine. Contrary to our findings, both studies found a weak association between age and baseline CGRP concentration. However, we must point out that our population consisted mostly of subjects below 30 years of age with only episodic migraine.

Lee et al. (24) found no elevated CGRP levels in patients with chronic migraine and no association with number of headache days, severity of attacks or headache on the day of blood sampling. Our result support the assumption that CGRP is neither associated with the number of monthly headache days nor monthly migraine days.

There is only limited data on CGRP measurements in human regarding migraine with and migraine without aura. As cortical spreading depression results in a significant depolarization, one can expect an influence on numerous neuronal interactions directly influencing the release of neurotransmitters and neuromodulators. Exemplarily, the expression of cortical CGRP mRNA was induced by repetitive CSD in mice 24 h following stimulation (25, 26). Taking this into account, it is conceivable that CGRP levels differ mainly in the postictal phase between patients with and without aura. This might explain the inconsistent results of studies investigating CGRP levels between those two groups.

Correct sampling of blood including the exact time point of sampling is also important when considering sex and gender influences on CGRP. An association of sex hormones (particularly estrogen, progesterone, and their interaction) with migraine is largely known. Recent studies provided evidence that CGRP levels are also modulated by these hormones (27). Therefore, to provide reliable data on CGRP differences between female and male subjects, plasma levels of sex hormones would be required.

To summarize, there is far too few data and too many heterogenous sampling and analysing methods regarding CGRP levels in human to compare results from different studies. Our study, however, bypasses the individual factors by longitudinal measurements of CGRP levels mainly depending on hypoxic stimulation. Hence, changes within groups and subgroups can be interpreted to be associated with the hypoxic challenge.

### Limitations

The findings in this report are subject to at least four limitations. First, the sample size with 30 subjects is small but comparable to other studies (23). However, we are aware that some subgroup analyses are based on a few participants only—and therefore cannot be examined for possible confounders such as gender or age. Second, a headache or migraine attack was “artificially” triggered in subjects using a hypoxic challenge. Utilizing a well-established migraine model minimizes confounders like hormones, nutrition, prophylactic, acute medication, or vague onset of the migraine attack. However, hypoxia cannot be fully ruled out as a confounder. Therefore, our results may not be applicable to unprovoked attacks. Third, our study is missing a control group. Initially, the study was designed using an active control, since we only expected inducing a migraine attack in 50% of the subjects. As mentioned elsewhere (21), a blinded control group is not feasible in a high-altitude chamber trial. Fourth, as this is a pilot study, we have chosen a very conservative statistical approach using Greenhouse-Geisser correction for violation of sphericity in repeated measures ANOVA. Still, our results are significant, which gives strength to our data.

A positive aspect of the study is the harmonized and standardized implementation of the experiment and the examinations in a homogenous patient population. As all subjects entered the NHC at the same time of the day, possible cycling variations of CGRP were minimized.

Taken together we could show significant different baseline levels in migraineurs, with reliable fluctuations during a provoked migraine attack. Since our measurements were done with a commercially available ELISA following a strict published protocol, we believe that our study could serve as a benchmark for future investigations.

## Data Availability Statement

The original contributions presented in the study are included in the article, further inquiries can be directed to the corresponding author.

## Ethics Statement

The study was reviewed and approved by the Ethics Committee of the Medical University of Innsbruck, Austria. The patients/participants provided their written informed consent to participate in this study.

## Author Contributions

FF: investigation, methodology, conceptualization, data curation, formal analysis, validation, and writing—original draft preparation. KK: investigation, conceptualization, data curation, formal analysis, and original draft preparation. KM: data analysis, methodology, supervision, and writing—review and editing. GB: conceptualization, methodology, validation, supervision, and writing—review and editing. All authors contributed to the article and approved the submitted version.

## Funding

This research was supported with a DOC Fellowship of the Austrian Academy of Sciences.

## Conflict of Interest

The authors declare that the research was conducted in the absence of any commercial or financial relationships that could be construed as a potential conflict of interest.

## Publisher's Note

All claims expressed in this article are solely those of the authors and do not necessarily represent those of their affiliated organizations, or those of the publisher, the editors and the reviewers. Any product that may be evaluated in this article, or claim that may be made by its manufacturer, is not guaranteed or endorsed by the publisher.
